# Outcomes and challenges of a kidney transplant programme at Groote Schuur Hospital, Cape Town: A South African perspective

**DOI:** 10.1371/journal.pone.0211189

**Published:** 2019-01-25

**Authors:** Bianca Davidson, Tinus Du Toit, Erika S. W. Jones, Zunaid Barday, Kathryn Manning, Fiona Mc Curdie, Dave Thomson, Brian L. Rayner, Elmi Muller, Nicola Wearne

**Affiliations:** 1 Department of Nephrology and Hypertension, Groote Schuur Hospital, University of Cape Town, Western Cape, South Africa; 2 Kidney and Hypertension Research Unit, University of Cape Town, Western Cape, South Africa; 3 Department of Surgery, University of Cape Town, Western Cape, South Africa; Imperial College Healthcare NHS Trust, UNITED KINGDOM

## Abstract

**Introduction:**

Access to dialysis and transplantation in the developing world remains limited. Therefore, optimising renal allograft survival is essential. This study aimed to evaluate clinical outcomes and identify poor prognostic factors in the renal transplant programme at Groote Schuur Hospital [GSH], Cape Town.

**Method:**

Data were collected on all patients who underwent a kidney transplant at GSH from 1st July 2010 to the 30 June 2015. Analyses were performed to assess baseline characteristics, graft and patient survival, as well as predictors of poor outcome.

**Results:**

198 patients were transplanted. The mean age was 38 +/- 10.5 years, 127 (64.1%) were male, and 86 (43.4%) were of African ethnicity. Deceased donor organs were used for 130 (66.7%) patients and living donors for 65 (33.3%). There were > 5 HLA mismatches in 58.9% of transplants. Sepsis was the commonest cause of death and delayed graft function [DGF] occurred in 41 (21.4%) recipients. Patient survival was 90.4% at 1 year and 83.1% at 5 years. Graft survival was 89.4% at 1 year and 80.0% at 5 years. DGF (HR 2.83 (1.12–7.19), p value = 0.028) and recipient age > 40 years (HR 3.12 (1.26–7.77), p value = 0.014) were predictors of death.

**Conclusion:**

Despite the high infectious burden, stratified immunosuppression and limited tissue typing this study reports encouraging results from a resource constrained transplant programme in South Africa. Renal transplantation is critical to improve access to treatment of end stage kidney disease where access to dialysis is limited.

## Introduction

Over the last two decades early kidney transplantation outcomes have improved dramatically due to better immunosuppression, enhanced understanding of immunology and advances in technical approaches.[1] Transplantation remains the treatment of choice for end stage renal disease [ERSD] due to superior survival rates, better quality of life and cost saving. [1–4] Transplantation in the public sector in South Africa [SA] is a vital service since dialysis is often rationed due to limited resources. The Western Cape Provincial Government has formally adopted a priority setting policy for acceptance onto dialysis, which can be defended, ethically and legally.[5, 6] In many state facilities a new patient can only be offered chronic dialysis when an existing patient is successfully transplanted. Therefore, transplantation is essential to provide access to new patients requiring renal replacement therapy [RRT].

Despite the known benefits, transplantation in Sub-Saharan Africa [SSA] has unique challenges and is limited in scope. The evolving epidemic of communicable diseases particularly human immunodeficiency virus [HIV] and tuberculosis [TB] [7] and an ever-increasing burden of non-communicable diseases [8], fuels the increasing incidence of chronic kidney disease [CKD]. This problem is compounded by limited numbers of nephrologists[9], limited resources, poor access to RRT[10] and a high burden of poverty. Most of SA’s population accesses public-sector medical care with many countries in SSA having no public sector access at all.[11–13] In Africa, it is reported that only 16% of patient requiring RRT receive it.[10] As a result of these challenges transplantation in SA falls into the lowest quartile of transplantation rates worldwide, with less than 10 per million population. [1]

SA remains one of only 12 countries within Africa that perform renal transplantation, and the only country in Africa that relies on deceased donation for the majority of its transplants.[14] Unfortunately the number of deceased donors has declined over the last two decades, necessitating a more liberal approach to donor selection allowing the service to expand the deceased kidney donor pool. This includes a HIV positive-to-positive transplant program, the utilisation of extended criteria donors [ECD] as well as donors after circulatory death [DCD].[15, 16]

Groote Schuur Hospital [GSH] is a public academic hospital in the Western Cape Province in SA that serves an estimated population of 6,362,257 million. Around 75% of this population is uninsured and therefore relies on public sector medical care. [11, 12] Acute dialysis is free for indigent patients. However chronic dialysis is rationed and limited to 148 slots (98 for haemodialysis and 50 for peritoneal dialysis). This process is ethically endorsed and strictly adhered to.[6, 17] A study by Kilonzo et al reviewing the selection criteria, reported that of the 569 patients presented for the RRT in a four-year period, more than half 53.9% were not accepted.[6] The selection criteria for acceptance are primarily based on suitability for transplantation.[6] The programme also provides transplantation services for patients from elsewhere in the Western Cape (George), the Eastern Cape (East London and Port Elizabeth) and the Northern Province (Kimberley).

To date there is very little information reporting outcomes on transplantation programmes using deceased donation in low middle income countries [LMICs] and African countries.[13] Therefore this study aimed to evaluate transplant outcomes and identify poor prognostic factors in a resource-limited setting.     

## Methods

This study was an observational cohort study. The data were collected on all patients who underwent kidney transplant at GSH from 1st July 2010 to the 30 June 2015. Ethical approval for the study was received from the human research ethics committee at University of Cape Town [HREC: 759/2014]. This approval permitted a folder review of patients transplanted within this time period. Informed consent for folder review was waived. All clinical and laboratory data were obtained from routine clinic visits which followed the best clinical practice guidelines set out by KDIGO.[18] All kidney biopsies were only performed for clinical indications including unexplained haematuria, proteinuria or rising creatinine. All patients were anonymised prior to statistical analysis. None of the transplant donors were from a vulnerable population and all donors or next of kin provided written informed consent that was freely given. The primary objectives of the study were to determine patient and graft survival estimates. The secondary objectives were to assess the factors associated with poor outcomes.

The data collected on the recipients included demographics (age, sex, race); baseline status (comorbidities, mode of dialysis, cause of ESRD, and previous transplantation). Immunological risk assessment included (panel reactive antibody assay [PRA] and human leucocyte antigen [HLA] mismatching). The donor details were also recorded: type of donor (living donor [LD] or deceased donor after brain death [DBD] or deceased donor after circulatory death [DCD]), sex, creatinine and cause of death in deceased donors.

The details of the transplant were collected: number of previous transplants, cold ischaemic time, length of stay in hospital, complications and delayed graft function [DGF]. Follow-up clinical data of the recipients (creatinine, opportunistic infections, rejection episodes and graft outcomes) were collected from folder review from routine clinical follow-up.

A Rutherford-Morison incision was used to facilitate extra-peritoneal graft placement. During deceased donor transplants, the renal artery was anastomosed using a Carrel patch. Extension of the right renal vein with donor inferior vena cava was performed at the surgeon's discretion. Traditionally, kidneys retrieved from living donors were anastomosed to the internal iliac artery in an end-to end fashion. However, during the last 18 months of the study period, end-to side anastomosis to the external iliac artery was preferred. Urinary tract continuity was re-established by anti-reflux uretero-neocystostomy (extravesical Lich Gregoir technique). Uretero-ureterostomy was performed in the presence of short ureters or inaccessible bladders. Double-pigtail ureteric stents (Fr6) were used in patients with abnormal bladders

Rejection episodes peri-transplant and within the study period were collected. Rejection was classified according to the BANFF 2007 criteria. Renal biopsies were performed when clinically indicated.

The type of immunosuppression was documented. Induction therapy, continuation therapy and reason for treatment change were recorded. Treatment options were stratified based on immunological risk as per the institute’s protocol. Low risk patients (low PRA <30% and not previously transplanted) received no induction and were started on cyclosporine, corticosteroids and azathioprine. Patients were categorised as high risk if they had donor-specific antibodies [DSA], high PRAs (>30%), previous transplantation or if HIV positive. These patients received induction therapy with anti-thymocyte globulin [ATG] and were initiated on tacrolimus, corticosteroids and mycophenolate mofetil [MMF]. In 2014, Basiliximab became available to our programme and was added to our protocol for patients with intermediate risk in conjunction with cyclosporine and azathioprine. For full details of the GSH unit’s transplant protocol see S1 File. Due to individual side effect profiles particularly for gout and cosmetic concerns in females, cyclosporine was switched to tacrolimus in selected patients.

Isoniazid prophylaxis was given for 1 year and valgancyclovir for 3 months. If ATG was used as induction then cotrimoxazole was given for 6 months and the duration of valgancyclovir was extended to 6 months.

During the study period HLA typing was initially limited to HLA A, and B. Class 2 HLA antibody testing was only commenced in 2013 and included DR and DQ loci. A CDC T-cell crossmatch was performed on all deceased donor transplants and a flow crossmatch was only performed on LD who had a DSA. B-cell crossmatching was not performed because of limitations in the tissue-typing lab. Solid phase based Luminex assay screening for antibodies with mean fluorescent index [MFI] flow cytometry was only available from 2013. Desensitization (plasma exchange with intravenous immunoglobulin) was performed on all LDs with a positive flow crossmatch.

For patients with more than one transplant, outcome analyses were based on the later graft. Graft failure was defined as patients requiring permanent RRT, graft removal or re-transplantation. DGF was defined as renal dysfunction and oliguria requiring dialysis within the first week of transplantation. Primary graft failure [PGF] was defined as a graft that perfused intra operatively but never functioned. Patients who died were sub-divided into those with or without a functional graft. Details on cause of death were obtained from hospital records, death certificates and contacting family members.

### Statistical analysis

Baseline patient characteristics, clinical variables and donor population were described using summary statistics. Continuous variables were summarised as mean (± standard deviation [SD]) or median (with interquartile range [IQR]) depending on the distribution of data. Categorical variables were summarised as frequency and percentages.

The primary outcomes of interest were time to death and time to death-censored graft survival. Patients who did not experience either outcome were censored at last follow up or end of study period (30 June 2015). Survival probability at 1, 2 and 5 years was estimated using Kaplan-Meier method, and associations between risk factors and primary outcomes (time to death or time to death-censored graft failure) were analysed using univariable and multivariable Cox proportional hazards models. Variables included in survival models were chosen *a priori* based on their clinical relevance (recipient age, DGF, rejection episodes, donor type and donor age) or to control for possible confounding (age, gender and race). Episodes of rejection were included in models as a time varying covariate [tvc]. HRs were reported with 95% confidence intervals [CI] to increase interpretation of precision of estimates. None of variables included in final multivariable models violated the proportional hazards assumption. All analyses were conducted using Stata software, version 14.2 (StataCorp, College Station, Texas, USA).

Reporting of results were reported according to STROBE (Strengthening the Reporting of Observational studies in Epidemiology) guidelines.

## Results

198 renal transplants were performed between 1^st^ July 2010 and the 30^th^ June 2015.

### Donor population

In the study, 130 (66.7%) patients received kidneys from deceased donors and 65 patients (33,3%) from living donors. The unit has a small DCD programme resulting in 7 donations during the study period. 11 patients were enrolled in the HIV positive donor to HIV positive recipient trial. Trauma accounted for 67 (54%) of deceased donations, the vast majority being male 56 (84%). Females accounted for 36 (59%) of LDs. Table 1 describes the demographic breakdown of the donor population.

**Table 1:** Demographic baseline characteristics of our donor population.

		Living Donors	Deceased donors (n = 130)		
		LD (n = 65)	DBD (n = 123)	DCD (n = 7)	P—values
Age*	Med [IQR]	35 [28–41]	31 [23–43]	34 [21–46]	0.279
Gender: Male:*	n (%)	25 (41%)*	82 (67%)*	6 (86%)	0.001
Race:*					0.168
Mixed Ancestry	n (%)	27 (45%)*	53 (43%)*	2 (29%)	-
African	n (%)	19 (32%)*	33 (27%)*	5 (71%)	-
Caucasian	n (%)	14 (23%)*	36 (30%)*	0 (0%)	-
**Creatinine**	Mdn [IQR]	74 [63–86]	92 [76–124]	80 [73–152]	0.001
**Cause of death:**					0.019
**Medical**	n (%)	-	56 (46%)	0	-
**Trauma**	n (%)	-	67 (55%)	7 (100%)	-
**Cold Ischaemic Time (Hrs.)**	Med (IQR)	4 [2–4]	10 [5–16]	14 [8–17]	<0.001

(DBD) Donor after Brain Death, (DCD) Donor cardiac death, [LD] Living donor.

* n varies due to missing data, denominators may vary from total cohort shown above

### Recipient population

Table 2 describes the baseline characteristics of the recipients. 198 patients were transplanted during the study period. For 181 (94%) of patients this was their first transplant. The mean age was 38±10.5 years. The recipients were predominantly male 127 (64.1%). The ethnicity of the cohort consisted of 96 (48.4%) mixed ethnicity and 86 (43.4%) black African race. Thirteen recipients were HIV positive and 11 patients were enrolled in the HIV positive donor to HIV positive recipient trial. Hypertension and chronic glomerulonephritis were the leading causes of ESRD, 72 (38.5%) and 65 (34.8%) respectively. (Table 3)

**Table 2:** Baseline characteristics of transplant recipients.

Demographics		Recipients (n = 198*)	LD* (n = 65)	DBD/ DCD* (n = 130)	P- Value
**Age (years)**	**Mdn [IQR]**	39 [30–46]	37 [29–43]	39 [31–47]	0.222
**Gender: Males**	**n(%)**	127 (64.1)	39 (60.0)	87 (66.9)	0.341
**Race:** Black African	**n(%)**	86 (43.4)	20 (30.8)	65 (50.0)	<0.001
Mixed Ethnicity	**n(%)**	96 (48.4)	32 (49.2)	62 (47.7)	-
White	**n(%)**	16 (8.1)	13 (20.0)	3 (2.3)	-
**First Transplantation**	**n(%)**	181 (91.4)	59 (90.8)	119 (91.5)	0.858
**HIV positive patients**	**n(%)**	13 (6,6)	0 (0)	13 (10)	-
Dialysis mode:* Haemodialysis	**n(%)**	141 (74.6%)	37 (59.7)*	102 (81.6)*	<0.001
Peritoneal dialysis	**n(%)**	44 (23.3%)	21 (33.9)*	23 (18.4)*	-
Pre-emptive	**n(%)**	4 (2.1%)	4 (6.5)*	0 (0)*	-
Donor HLA mismatches*: 5–8	**n(%)**	93 (58.9)*	16 (32.7)*	77 (71.3)*	<0.001
PRA >30 at time of transplant*	**n(%)**	29 (17.3)*	10 (10.8)*	19 (16.8)	0.745
**Length of stay (days)**	**Med [IQR]**	13 [10–18]	13 [11–16]	14 [10–20]	0.684

(DBD) Donor after brain death, (DCD) Donor cardiac death, [LD] Living donor, (HLA) Human Leucocyte Antigen, (PRA) Panel Reactivity Antibody Assay.

* n varies due to missing data, where denominator will be less than that stated above

**Table 3:** Causes of ESRD in renal transplant recipients (n = 189).

Cause	Number	Percentage
Hypertension	72	38.5%
Chronic Glomerulonephritis	65	34.7%
Familial Kidney disease	11	5.9%
Diabetes	11	5.4%
Unknown	7	3.7%
HIVAN	6	3.2%
Obstructive uropathy	7	3.2%
Other	5	2.7%
SLE	4	2.1%
Rapidly progressive Glomerulonephritis	1	0.5%

(HIVAN) Human Immunodeficiency Virus Associated Nephropathy, (SLE) Systemic Lupus Erythromatosis

### Transplantation period

**Fig 1:**
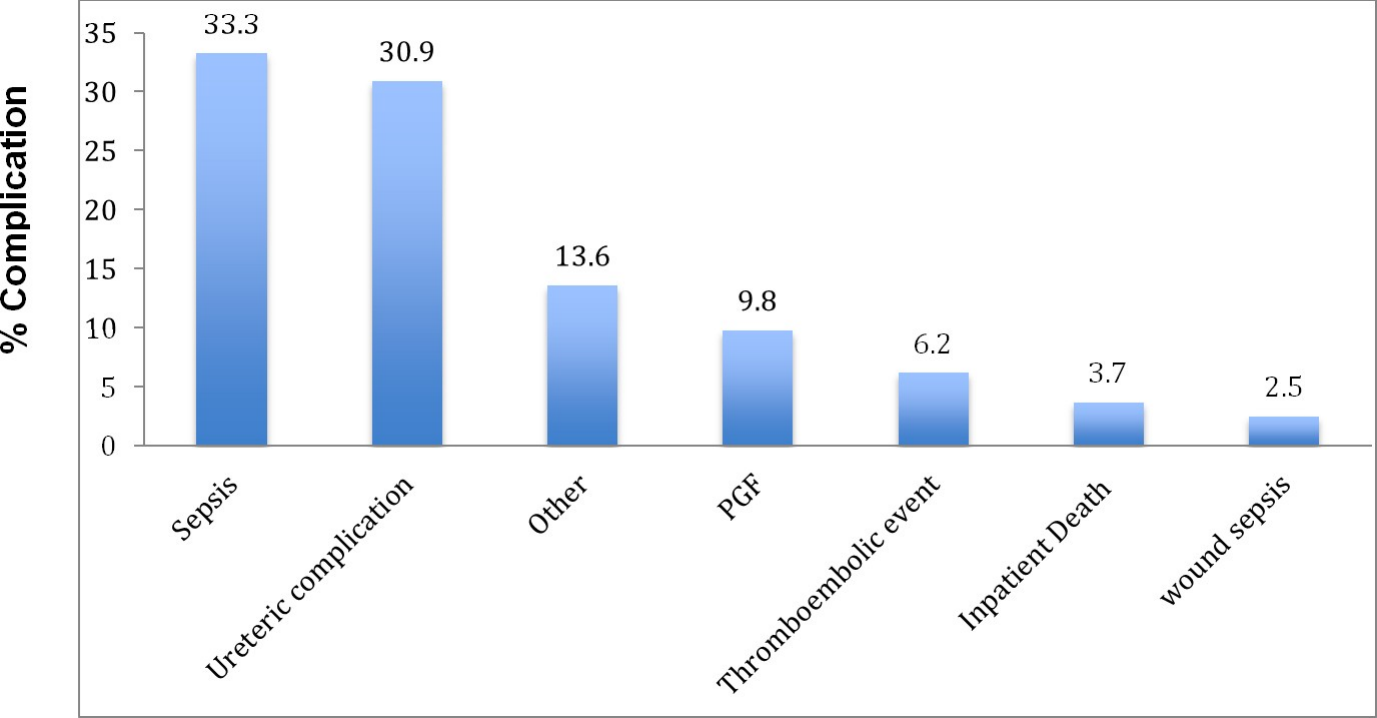
Bar graph demonstrating post-transplant complications.

The median cold ischaemic time was 4 hours [IQR 2 – 4hrs] for LDs and 10 hours [IQR 5 – 16hrs] in deceased donors. The median hospital stay was 13 days [IQR 10–18] with no difference seen between donor types. Of the recipients only 29 (17.3%) had a PRA of greater than 30. HLA matching is not a priority in our unit therefore most patients 77 (71.3%) had > 5 mismatches. Induction therapy and initial immunosuppression received is shown in Table 4. DGF occurred in 41/192 (21.4%) of the transplanted patients. Fig 1 describes the post transplant complications. The most common perioperative complication was sepsis followed by ureteric complications (ureteric stenosis n = 13, urine leak n = 10, vesico-enteric fistula n = 1). Prior to discharge 35 patients required a medication change. The reasons for this included rejection (n = 22), side effects (n = 9) and interstitial nephritis (n = 4).

**Table 4:** Treatment regiments used in the cohort.

Induction and initial immunosuppression used		
AZA + CYA	**n(%)**	95 (51%)
AZA + CYA + Basiliximab	**n(%)**	18 (10%)
Tacrolimus + MMF	**n(%)**	30 (16%)
Tacrolimus + MMF + ATG	**n(%)**	40 (22%)
Tacrolimus + MMF + basiliximab	**n(%)**	2 (1%)
Alternative regiment	**n(%)**	11 (4%)

(AZA) Azathioprine, (CYA) cyclosporine, (MMF) Mycophenolate Mofetil, (ATG) Antithymocyte Globulin. Alternative regiment: Tacrolimus + AZA, MMF + cyclosporine with +/- induction therapy.

### Rejection

Table 5 reflects the biopsy proven rejection episodes seen within the first month, in the first year and between 1–5 years post transplantation. Within the first month we had 32 rejection episodes, and at the end of the first year there was a total 59 episodes. Rejection reported within this first year included repeat events. Between the end of the first year and 5 years there were only 11 episodes. There were 11 cases that were not biopsy proven and treated on the basis of clinical suspicion. These cases have not been included in the rejection analysis.

**Table 5:** Biopsy proven rejection occurring within the study cohort.

Biopsy proven rejection episodes (n = 198 recipients)				
		First Month (n = 32)	1 month—1 yr. (n = 27)	> 1yr– 5 yrs. (n = 11)
**Cellular rejection**	n(%)	7 (20.6%)	10 (37.0%)	2 (18.2%)
**ABMR**	n(%)	11 (34.4%)	3 (11.1%)	4(36.4%)
**Cellular + ABMR**	n(%)	3 (9.4%)	1 (3.7%)	-
**Borderline rejection**	n(%)	11(34.4%)	13 (48.1%)	5 (45.5%)

(ABMR) Antibody mediated rejection

### Patient and graft survival

**Fig 2:**
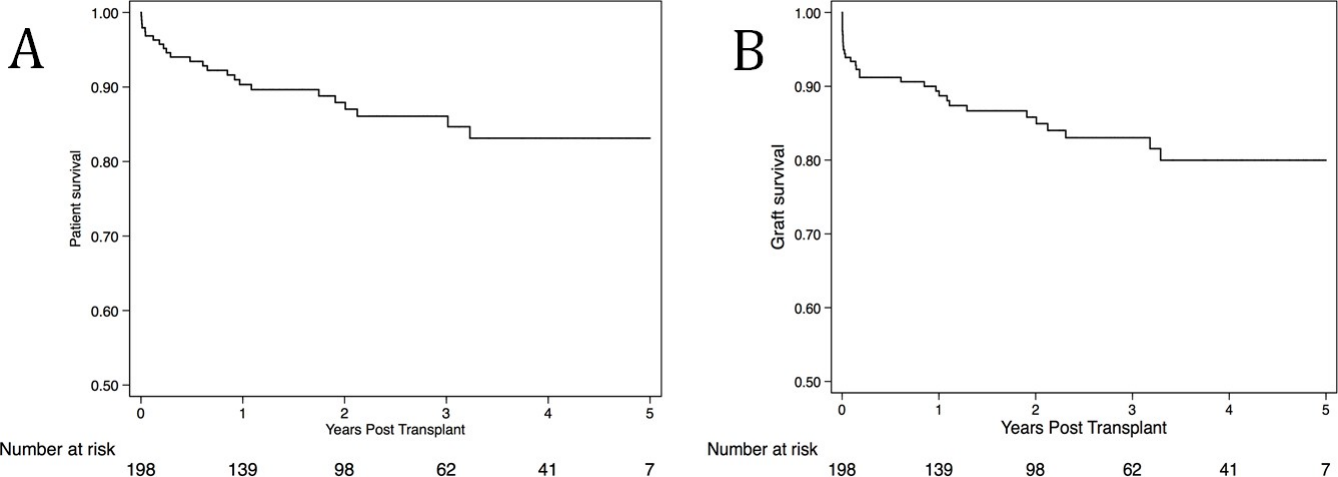
Kaplan Meier curves showing A) Patient survival and B) Death censored Graft Survival.

For entire cohort: Patient survival at 1, 2 and 5-years was 90.4% [95% CI 84.9–93.9%], 87.9% [95% CI 81.8–92.1%] and 83.1% [95% CI 75.1–88.8%] respectively. Our graft survival (censored for death with a functional graft) at 1, 2 and 5 year was 89.4% [95% CI 84.0–93.0%], 85.8% [95% CI 79.6–90.3%] and 80.0% [95% CI 71.8–86.0%] respectively. Fig 2 demonstrates Kaplan-Meier patient and graft survival curves. Twenty-four patients died during the study period.

Survival per donor type: Living donor patient survival at 1-, 2- and 5-years was 93.3% [95% CI 82.9–97.5%], 88.2% [95% CI 75.0–94.7%] and 85.0% [95% CI 70.0–92.8%] and survival from deceased donation was 89.1% [95% CI 81.8–93.5%], 87.8% [95% CI 80.2–92.6%] and 84.5% [95% CI 75.3–90.5%] respectively. Graft survival for transplants from living donation at 1-, 2- and 5-years was 90.3% [95% CI 79.6–95.5%], 85.8% [95% CI 73.3–92.7%] and 82.6% [95% CI 68.5–90.9%] and deceased donation was 88.8% [95% CI 81.7–93.2%], 85.6% [95% CI 77.7–90.9%] and 78.6% [95% CI 67.8–86.1%] respectively.

The causes of death included sepsis (n = 13), cardiovascular events (n = 2), malignancy (n = 2), thromboembolic events (pulmonary embolism and cerebrovascular event) (n = 2), post-surgical haemorrhage (n = 1), and 1 from rejection where dialysis support was not offered. In 3 patients the cause of death was not identified. Twelve patients died with a functioning graft. The most common cause of death in the 24 patients was sepsis 13 (54.2%). Table 6 details the aetiology of the septic episodes and timing in relation to transplantation.

**Table 6:** Detailing the aetiology of the deaths caused by sepsis.

Patient	Time of death since Transplant	Aetiology of sepsis	Summary
1	**> 1 year** Died at 2 years post transplant	Died from pyelonephritis (fungal). Not for RRP due to non-compliance therefore not for ICU. Died in renal failure with sepsis.	Not a candidate for ICU as declined RRP. Died of sepsis.
2	**<1 year** Died at 11/12	Not for RRP (non compliant). Presented with acute T-cell rejection, despite treatment of rejection, no improvement in kidney function. ESRF on biopsy. Not for RRP therefore not for ICU. Died of sepsis (*Staphlococcus aureus*) in unit.	Not a candidate for ICU as declined RRP. Died of sepsis.
3	**>1 year** Died at 2 years post transplant	Noted to have compliance issues. Had prior episode of acute rejection at 8 months. Creatinine continued to rise despite treatment of acute cellular rejection. Repeat biopsy showed CAN and ESRF. Presented with *Escherichia coli* sepsis from urinary tract infection.	*Escherichia coli* sepsis 2 years post transplant. Died of sepsis.
4	**<6/12** Died 2/12 post transplant	Multiple complications post transplantation. Post operative period complicated by urinary leak. Then developed a nosocomial pneumonia, bronchoscopy revealed *Pseudomonas aeruginosa* on sputum culture. Overwhelming sepsis with no response to treatment in ICU on full support including ventilation and dialysis.	Overwhelming sepsis post transplant. Nosocomial *Pseudomonas aeruginosa* pneumonia and AKI. Died in ICU < 6/12 post transplant
5	**>1 year** Died at 3 years post transplant	Initial event was a cellulitis, which was treated in hospital. Developed a nosocomial sepsis with *Acinetobacter baumannii* (blood culture). Admitted to the ICU, demised despite full support.	Nosocomial sepsis following admission for cellulitis. 3 years post transplant
6	**< 6/12** Died 2/12 post Transplant	Multiple complications post transplantation. Recurrent *Acinetobacter baumannii* and *Klebsiella pneumoniae* urinary tract infection. *CMV* viraemia treated. Admitted to ICU, 2/12 post discharge from the transplant unit. Died 5/7 later with *Klebsiella pneumoniae* septicaemia cultured on blood culture.	Overwhelming sepsis post transplant. *Klebsiella pneumoniae* septicaemia. Died in ICU <6/12 post transplant
7	**< 6/12** Died 5/12 post transplant	Multiple complications post transplantation. Biopsy at 5/12 showed borderline rejection and the immunosuppression was escalated. This was complicated by *Enterobacter* sepsis and *CMV* viraemia. Fluid collection around graft complicated by graft failure. Wound sepsis, with septicaemia died in ICU	Overwhelming sepsis post transplant. Nosocomial *Enterobacter* sepsis + *CMV*. Died in ICU < 6/12 post transplant.
8	**< 6/12** Died 15/7 post transplant	Wound sepsis 5/7-post transplantation. *Escherichia coli* cultured in deep wound collection. Complicated by nosocomial urine tract sepsis with *Klebsiella pneumoniae*. Transferred to ICU with overwhelming sepsis, died in ICU.	Overwhelming sepsis post transplant. Nosocomial *Klebsiella pneumoniae* sepsis. Died in ICU < 6/12 post transplant.
9	**< 6/12** Died 49 days post transplant	Admitted with nosocomial pneumonia 49 days post transplantation. Cultured ESBL *Enterobacter*. Died in ICU despite full support.	Overwhelming sepsis post transplant. Nosocomial ESBL *Enterobacter* sepsis. Died in ICU < 6/12 post transplant.
10	**< 6/12** Died 2/12 post transplant	Multiple complications post transplantation. Initially had wound sepsis (*Staphlococcus aureus*). Nosocomial sepsis with *Acinetobacter baumanni* on blood culture. Complicated by nosocomial pneumonia, which cultured *Klebsiella pneumoniae* and *Pseudomonas aeruginosa*. *CMV* viraemia. Overwhelming septicaemia. Died in ICU.	Overwhelming sepsis post transplant. Nosocomial *Acinetobacter baumanni*, *Klebsiella pneumonia*, Pseudomonas *aeruginosa* and *CMV*. Died in ICU <6/12 post transplant.
11	**> 1 year** Died at 1yr post transplant	Appendectomy in October 2014. Intra-abdominal collection complicated with necrotic bowel. Complicated with nosocomial sepsis with *Escherichia coli* and *Enterococcus* septicaemia and death in the ICU.	Nosocomial sepsis following a complication post appendectomy > 1 yr.
12	**< 6/12** Died 2/12 post transplant	Multiple complications post transplantation. Complication included delayed graft function and wound dehiscence. Post discharge developed ABMR, immunosuppression escalated. Patient developed MDR TB in the Eastern Cape and died.	Died 2/12-post transplant of MDR TB post escalation of immunosuppression.
13	**> 1 year** Died at 3yr post transplant	Noted to be non-compliant (low tacrolimus level). Now with ESRF due to rejection. Died from *Escherichia coli* sepsis from urinary tract infection.	Not a candidate for ICU as declined RRP. Died of sepsis.

(ICU) intensive care unit, (RRP) renal replacement programme, (ESRF) end stage renal failure, (CAN) chronic allograft nephropathy, (CMV) cytomegalovirus, (ESBL) Extended Spectrum Beta-Lactamases, (ABMR) Antibody mediated rejection, (MDR TB) Multidrug resistant Tuberculosis

The PGF rate was 8/198 (4%). Our 30-day graft nephrectomy rate was 11/198 (5.5%). Eight patients (4%) underwent graft nephrectomy for acute vascular thrombosis (6 arterial and 2 venous), as the grafts were not salvageable on re-exploration. Five of the six arterial thromboses occurred in living donor recipients and two were venous thromboses. Three additional graft nephrectomies were performed due to urological complications, which resulted in graft failure.

Table 7 demonstrates factors associated with mortality and graft failure on multivariate analysis. The following factors were found to be associated with poor patient survival in the multivariate analysis: Recipient age (HR 3.12, 95% CI = 1.26–7.77, p = 0.014) and DGF (HR 2.83, 95% CI = 1.12–7.19, p = 0.028). There were no statistically significant associations in graft failure in the multivariate model.

**Table 7:** Factors associated with mortality and graft failure on multivariate analysis.

	Patient Survival		Graft survival	
Variable	HR (95% CI)	P value	HR (95% CI)	P value
Age > 40yrs	3.12 (1.26–7.77)	0.014	0.60 (0.27–1.35)	0.211
Gender (male)	2.65 (0.85–8.13)	0.094	-	-
Race (African)	1.80 (0.72–4.52)	0.213	1.78 (0.82–3.86)	0.147
DGF	2.83 (1.12–7.19)	0.028	0.62 (0.18–2.11)	0.441
Rejection episode	0.98 (0.36–2.69)	0.966	1.58 (0.59–4.23)	0.366
Donor Type (DBD/ DCD)	-	-	0.82 (0.35–1.93)	0.642
Donor Age > 40 yrs.	-	-	1.03 (1.00–1.06)	0.095

(DBD) Donor after brain death, (DCD) Donor cardiac death, (yrs.) years

## Discussion

This observational cohort study assessed the outcomes of a transplant programme in a resource-limited setting. In this cohort the most common cause of death was sepsis and the risk of death was higher in older patients and those who had DGF.

In the public sector, dialysis selection criteria favours younger, fitter, healthier patients as suitability for transplantation is the guiding principle to select patients for scarce dialysis slots.[19] Therefore recipients from our cohort differ from other established transplant databases. This is evident when comparing our results with well-established registries like the United States Renal Data System [USRDS] and Organ Procurement and Transplantation Network [OPTN].

Table 8 demonstrates patient and graft survival from large registries and from countries performing transplants with a similar gross domestic product [GDP] to SA. The first distinguishing difference is that the mean recipient age is 37 +/- 10.5 years. This contrasts the peak age bracket of transplantation in the USRDS of 45–64 years,[20] and furthermore in the OTPN, where the majority were > 50 years in the same age bracket.[21] Secondly our recipient cohort was healthier, with strikingly fewer patients with diabetes [5.4% compared to 34.5%].[20] Finally, in numerous published registry data the most common cause of death in transplant recipients was cardiac related.[20, 22, 23] In our cohort, sepsis was the leading cause of death.

**Table 8:** Comparison of transplant survival data.

Country	Date of data capture (n)	GDP/ capita ($)	DM (%)	Combined outcomes				Living related outcomes				Deceased outcomes			
				Patient Survival		Graft Survival		Patient Survival		Graft Survival		Patient Survival		Graft survival	
				1 yrs.	5 yrs.	1 yrs.	5 yrs.	1 yrs.	5 yrs.	1 yrs.	5 yrs.	1 yrs.	5 yrs.	1 yrs.	5 yrs.
ERA -EDTA [24]	2014 (n = 19406)		-	-	-	-	-	98.4	94.2	95.5	87.0	96.1	87.9	90.9	79.0
ANZ DATA [22]	2010–2014	51360.0 (Aus.) 38970.0 (NZ)	17.6	-	-	-	-	99.0	95.0	98.0	89.0	98.0	90.0	95.0	83.0
USRDS[25]	2014 (n = 17205)	58270.0	-	-	-	-	-	99.0	-	97.0	85.0	96.0	-	92.0	73.0
OPTN[26]	2008–2015	58270.0	-	-	-	-	-	98.5	92.0	97.5	85.6	96.2	83.1	93.2	74.4
Nigeria[27]	2010–2014 (n = 47)	2080.0	15.0	-	-	-	-	97.7	67.9	95.3	60.7	-	-	-	-
Morocco[28]	1998–2008 (n = 67)	2863.2	3.0	-	-	-	-	96.6	-	88.1	-	-	-	-	-
Brazil[29]	2007–2011 (n = 600)	8580.0	18.3	-	-	-	-	-	-	95.8	90.9	-	-	93.9	81.9
Tunisia[30]	1986–2015 (n = 702)	3500.0	2.0	96.0	89.3	95.0	86.5	-	-	-	-	-	-	-	-
Ivory Coast[31]	2012–2014 (n = 10)	1535.0	-	-	-	-	-	100.0	-	100.0	-	-	-	-	-
Libya[32]	2004–2005 (n = 50)	6540.0	-	-	-	-	-	96.0	-	94.0	-	-	-	-	-
Mexico [33]	1995–2003 (n = 1356)	8610.0	-	-	-	-	-	90.0	-	82.0	-	80.0	-	70.0	-
Mexico [34]	1976–1999 (n = 326)	8610.0	-	92.0	81.0	87.0	64,0	-	-	-	-	-	-	-	-
Pakistan[35]	1998–2010 (n = 3150)	1580.0	-	-	-	-	-	96.0	90.0	92.0	85.0	-	-	-	-
Austria[36]	1990–2013	45440.0	16.8	94.0	84.0	95.3	91.4	-	-	-	-	-	-	-	-
Cape Town	2010–2015 (n = 198)	5430.0	5.4	90.4	83.1	89.4	80.0	-	-	-	-	-	-	-	-

(ERA—EDTA) European Renal Association–European Dialysis and Transplantation, (ANZDATA) Australia and New Zealand Dialysis and Transplant Registry, (USRDS) United States Renal Data System, (OPTN) Organ Procurement and Transplant Network

The overall number in the study period was small (n = 198) compared to large registries that report outcomes. The majority (two thirds) of our cohort’s transplants were from deceased donation. When we analysed the outcomes in separate living and deceased groups the numbers were too small to make statistically meaningful interpretations. When comparing 5-year graft and patient survival outcomes to larger registries our combined patient survival was comparable to large deceased donor cohorts. This is despite numerous challenges including 1) stratified immunosuppression, 2) limited tissue typing availability 3) extreme poverty and 4) a high infectious burden, particularly TB and HIV.

DGF and a recipient age greater than 40 years had a statistically significant increased risk of death. DGF is known to impact on recipient and graft survival.[37, 38] Similarly, age has been identified as a major indicator of risk of death in the post transplant period, with 40–50 year old recipients having a relative risk of 2.3 when compared with recipients less than 40 years.[39]

Although not statistically significant, African race (HR 1.78) or an episode of rejection (HR 1.58) showed a moderate increased risk of graft failure on multivariate analysis. (Table 7) Black African race may serve as a proxy for other unmeasured factors such as poor socio-economic conditions.[40] Studies from the United States of America have found that African Americans had an increased risk of rejection and graft loss but not survival[41]; and demonstrated a higher rate of readmissions, which predicted worse renal outcomes. [42]

Sepsis was the leading cause of death in our cohort. This differs from other large registry data, which describes cardiovascular events as the leading cause of death. Our high burden of sepsis needs to be interpreted in the context in which we practice. In 2017, 7.06 million people were documented to be HIV positive in SA with a staggering estimated TB incidence of 438000 in 2016. This is coupled with poverty, malnutrition and resource rationing in our hospital especially for high care and intensive care unit [ICU] support. Thirteen patients died of sepsis. Three presented with sepsis and ESRD and were declined renal replacement and intensive care support due to non-compliance. Seven died within 6 months of transplantation, all experienced multiple episodes of sepsis in the postoperative period and died in ICU from nosocomial septicaemia. Of the patients that died 8 (61.5%) died within the first year of transplantation. See Table 6

Our programme also differs from high-income countries in that, due to cost constraints, we utilise immunological risk stratification protocols to determine treatment regimens. This is discussed as an alternative in the KDIGO clinical practice guidelines which states “if drug costs block access to transplantation, a strategy to minimise drug costs is appropriate, even if use of inferior drugs is necessary to obtain the improved survival and quality of life benefits of transplantation compared with dialysis.”[43] This strategy for low risk patients includes limited use of biologic agents for induction, the use of azathioprine in preference to MMF and cyclosporine as the calcineurin inhibitor of choice.

We report 59 episodes of rejection at the end of the first year within our cohort of 198 patients. This high rejection rate is multifactorial. It is important to acknowledge the limitations in the available tissue typing offered during the study period. From 2010 to 2013 we were only performing and measuring class 1 HLA Antibodies. Anti class 2 HLA Antibody testing was only available from 2013 onwards. B cell cross matches are still not available. Single Luminex antigen testing was only introduced for patients awaiting deceased donation on the transplant waiting list after the study was completed.

In 6 cases of PGF, venous thrombosis was identified as the cause of graft loss. A proportion of our dialysis population had prolonged femoral catheters in femoral veins for dialysis, which is a risk factor for future graft loss.[44] Arterial complications could be related to immunological risk factors. All arterial anastomosis were done in a standardised fashion i.e. anastomosing the renal artery to external iliac artery.

We recognise that this study has several limitations. Firstly, the study was performed in a single centre with retrospective study design. Secondly, the patient numbers between the living and deceased donor cohorts were too small to make meaningful statistical comparisons. In addition, no socioeconomic information was collected, which could have enhanced our inference on risk of death or graft failure in a lower socio-economic cohort. In view of our high septic complications this may prove to be important in the future when correlating graft and patient survival.

## Conclusion

SA is the primary transplanting centre in SSA. In a four-year period, we were able to transplant an equivalent number of patients to whom we can offer dialysis–effectively allowing us to “turn-over” our dialysis service in a 4-year time span. This provides life-saving treatment to those on the waiting list, who have no other option for RRT. Despite resource constraints, significant poverty, limited access to immunosuppression and tissue typing our survival outcomes at 5 years are comparable with larger registry data for deceased donors. Therefore, in a resource-constrained environment with limited access to RRT, transplantation still remains a viable and essential treatment option for CKD.

## Supporting information

S1 File GSH transplant protocol. (PDF)
