# Supplementary material for: Outcomes and challenges of a kidney transplant programme at Groote Schuur Hospital, Cape Town: A South African perspective
Source: PLoS One. 2019 Jan 25;14(1):e0211189. doi: 10.1371/journal.pone.0211189 (PMC6347365; doi:10.1371/journal.pone.0211189)
Supplement: S1 File — (PDF) [file pone.0211189.s001.pdf]

GSH Renal and Transplant Units

Kidney Transplant Protocol

Transplantation provides the best chance of a normal productive life to individuals with chronic renal failure. Optimum therapy requires a balance between sufficient immunosuppression to control rejection and little enough to avoid toxicity. All the currently available agents have various degrees of toxicity and optimum treatment requires the transplant physician to use both the available information as well as the available agents to tailor the prescription to optimize management for each individual. It is well recognized that many of the agents used are costly, and a variety of measures **may** be used to save costs. These are seldom of direct benefit to an individual patient but may be of benefit to a particular transplant program. The decision to make use of these cost savings measures should be made by the clinician and hospital administrators in conjunction. It is critical that sufficient flexibility is available to depart from any set protocol when that is seen to be in the best interests of the patient.

Most immunosuppressive protocols aim to use a combination of agents with differing mechanisms of action to achieve maximum effectiveness while minimizing side-effects. The protocol outlined below for transplantation drugs applies to renal transplantation only. This is the protocol to be currently used at Groote Schuur Hospital, and while we regard this as reasonable and cost-effective, other protocols may be considered more appropriate in other settings.

The IL2-Receptor Antagonist (IL2-RA) Basiliximab *(Simulect)* 20mg IV on day 0 and day 4 should be used in all patients as induction therapy to prevent acute rejection, except those receiving Anti-Thymocyte Globulin induction. Basiliximab can be omitted in HLA identical transplants.
All patients also receive Methylprednisolone (*Solumedrol*) 500mg day1, 250mg day2, 125mg day3, plus Prednisone 20mg/day. Wean Prednisone by 5mg/day every 2 weeks.
All living related transplant recipients should have their calcineurin inhibitor started 24-48 hours before the transplant.
Aspirin 150mg daily (3-5 days) should be started by the surgeons post-operatively, unless they feel the surgery was such that anti-coagulation would be unsafe.

1. De Novo Transplant-low immunological risk (HLA-identical transplants)

Cyclosporine(CyA) 3mg/kg bd (if used with Basiliximab induction) otherwise 4mg/kg bd (maximum initial dose 300mg bd), subsequently adjusted by trough blood concentrations - 200-300ng/mL in first 3 months, tapering to 150-250ng/mL till 1 year, and 75-200ng/mL thereafter. (ng/mL=ug/L).
Azathioprine (AZA). Start at 2mg/kg/day (max. dose 150mg daily) and continue, only reducing the dose in the face of leucopoenia (WCC<$4x10^9$/L). A bolus IV dose of 100mg is given intra-operatively.

2. Intermediate Immunological Risk Patients (all patients not Low or High Risk)

This intermediate risk strategy is not currently being implemented at GSH. See 1. De Novo Transplant-low immunological risk (HLA-identical transplants)
Cyclosporine(CyA) orally at 5mg/kg first dose and thereafter 2.5mg/kg bd, subsequently adjusted by trough blood concentrations - 100-200ng/mL (0-2months), 75-150ng/mL (2-4months), 50-100ng/mL thereafter. After 1 year, consider 25-50ng/mL
Everolimus at an initial dose regimen of 0.75mg bd should be administered as soon as possible after transplantation. Target trough blood concentrations are 3-8 ng/mL, but aim

for the lower end of this range especially in the early post-transplant period (to minimise wound healing issues and while CNI levels are high, thus minimising rejection risk).

3. High Immunological Risk Transplants

This is defined as patients who have:

1. rejected previous transplants
2. high preformed HLA antibody titres/panel reactive antibodies ≥30% (current/historic), though this % still needs to clearly defined using the new Luminex system.
3. HLA Donor-Specific HLA antibodies (DSA) ie. virtual crossmatch positive or even flow or CDC positive crossmatch positive in the absence of identifiable HLA DSAs.

Ideally all potential kidney transplant recipient's serum should be screened for donor specific antibodies. All potential recipients of a living related kidney transplant need Single Antigen Antibody Testing if the Multispecific/ID HLA Antibody test is at all positive (reflex testing has been implemented with the tissue typing laboratory). If these are present, transplantation should not proceed without first assessing the need for desensitization procedures (see 3.2 below), using flow crossmatch (FXM) results, which are currently not done by the GSH Tissue Immunology lab - send to NHLS Tissue Immunology lab in Johannesburg. These patients carry higher risk of rejection, even with a negative Compliment Dependent Cytotoxicity (CDC) cross-match and negative FXM, and should receive induction therapy (3.1):

<u>3.1 Induction therapy</u> with Anti-Thymocyte Globulin(eg. Fresenius ATG 2.5mg/kg/day - usually 200mg daily - *or* Genzyme Thymoglobulin 1.5mg/kg/day - rounded off to nearest 25mg) is administered at the time of surgery and subsequently administered based on the lymphocyte count ie. ATG should be omitted lymphocyte count is less than $0.2x10^9$/L. Administration continues for 3-5 days (aim for the total dose of Fresenius ATG to be 9mg/kg). The IL2-RA antibodies eg. Basiliximab (*Simulect)* may be used in place of ATG, but costs more and is less efficacious in truly high risk patients.
Patients receive in addition Tacrolimus, Prednisone and Mycophenolate (see below for details). This is because Tacrolimus[1,2] and Mycophenolate Mofetil[3] have been shown to decrease acute rejection and increase graft survival in the first year post transplant, so use in patients at known high risk of rejection seems logical, though there is no randomized data comparing this strategy to CyA/AZA based therapy in these patients at high risk of rejection.
In the event that the patient had experienced severe acute rejection with a previous graft while having adequate CyA blood concentration, then Tacrolimus and Mycophenolate should be used in place of CyA/AZA de novo. (see below).

<u>3.2 Desensitization procedures</u> for flow or CDC positive crossmatch transplants can involve plasmapharesis or IVIG (Polygam) or Rituximab or combinations of these. Rituximab, as a single dose of 375mg/m^2, is ideally used for all patients undergoing desensitization, and is given 2 to 4 weeks prior to the planned date for surgery (premedication with antihistamine and IV steroids as for ATG use). The magnitude of desensitization will depend on the number of DSAs and level of the mean fluorescent index (MFI), as well as the cross-match status. Transplantation cannot proceed unless the CDC cross match is negative. We recommend doing at least 3 sessions of alternate daily plasma exchange (PLEX). Aim to filter off 1.5x plasma volume - usually 3L to 4L, replacing the filtered fluid with 5% Albumin in a Balsol/PlasB solution, immediately prior

to transplantation (see 3.3.3 below for more details). If available (in a quick turnaround time), aim for at least a 50% decrease in the MFI of the DSA, otherwise the PLEX/IVIG should ideally be continued for longer. To prevent a rebound in antibodies, IVIG 100mg/kg should be given at the end of each PLEX session. This protocol has been shown to increase patient survival, as opposed to waiting on the list for a cadaver transplant[4]. If necessary, a further 2 sessions of PLEX can be performed post-transplantation (day 1 and 3). All patients receiving desensitization also receive ATG from the time of transplantation, plus Tacrolimus and Mycophenolate, as with all other high-risk patients (see above), ideally from the start of desensitization.

3.3 ABO-incompatible kidney transplant protocol

The key elements are:

1. Anti-A/B isohaemagglutinin titre measurement – done on a daily basis via the Blood Bank.

2. B-Cell depletion – Rituximab 375mg/m^2 given as a stat dose preferably 2-4 weeks before transplantation, but if this is not possible then **after** the first plasma exchange (PLEX) and IVIG (Polygam).

3. Antibody depletion – using conventional alternate daily PLEX as for HLA donor-specific antibody (DSA) desensitization ie. 1 to 1.5x plasma volume exchange ie. 3-4L, with low dose IVIG (100mg/kg) given after each PLEX – can be done over about 2 hours. The replacement colloid is with 5% Albumin in a Balsol/PlasB solution as in 3.2 above

   Estimated 1xplasma volume (in litres) = 0.07 x weight (kg) x (1 - haematocrit)
   Approximates 3.0L in patients of 60kg, 3.5L in patients of 70kg, 4.0L in patients of 80kg

Complications of PLEX include hypocalcaemia and clotting abnormalities, so Ca, INR and Fibrinogen should be measured regularly – at least alternate daily eg. on PLEX skip days, and replaced as needed.

The number of plasma exchanges is dependent on the initial Anti-A/B isohaemagglutinin titre measurement and is done alternate daily:

Initial titre	Number of planned PLEX/IVIG
<16	2
16-32	3
64	4
128	5-6
Desensitization is not recommended with titres of >128, but is possible:	
256	7-8
512	9-10
>512	>10

Transplantation should proceed within 24-48 hours after the Anti-A/B isohaemagglutinin titres drops to ≤8.

The last (anticipated) session of PLEX should be done with FFP (average 280+/-70mL per unit) making up 50% of the volume (donor or preferably AB blood group FFP) of the replacement colloid solution (and the other 50% albumin), as should the first session post-transplant if within 48 hours of surgery. IVIG must NOT be used with PLEX post-transplant because of the risk of inducing/worsening antibody mediated rejection (AMR)

caused by Anti-A/B antibodies in the IVIG. Blood transfusions (leucocyte-reduced packed cells), if needed, must be recipient blood group compatible.
Induction is with Basiliximab (20mg Day0 and Day4), unless there was an HLA DSA also present, in which case ATG should be used.
Steroids, Tacrolimus and MMF are used as for other high immunological risk patients with Tacrolimus and MMF/MPA started at the beginning of the desensitization protocol (at least 10 days before the transplant). Trough Tacrolimus levels are measured as per protocol – usually twice weekly and not before 48-72 hours after the first dose. Trough MPA levels should be measured if MMF-type side effects occur.
Anti-A/B isohaemagglutinin titre measurement continues daily post-transplant. If titres rise to 16 without any signs of rejection, this can be watched, but once they reach ≥32, then daily PLEX (without IVIG) should be restarted and continued daily until titres drop to ≤8 or until 2 weeks post-transplant. After 2 weeks, titres can be watched and allowed to rise, as long as there are no other signs of rejection – this phenomenon is called accommodation. Titres should continue to be measured on every clinic visit for the first 3 months, but AMR is unlikely after 2 weeks.
Biopsies should be performed at any sign of rejection and ideally a 1 month protocol biopsy should be performed as well. C4d positive staining on biopsy without any other signs of AMR is not unexpected (may be a sign of accommodation) and does not require treatment for rejection.
AMR that occurs later can be treated with PLEX/IVIG daily as above. Severe acute AMR that is resistant to this may require splenectomy.

4. Recipients of a Kidney from a Donor after Circulatory Death (DCD)

1. ATG (as above) within 2 hrs pre-op
2. Anti-metabolite as per immunological risk on day of surgery
3. Start CNI at usual dose only when:
    a. urine output >1.5L/day in previously oliguric patients or
    b. creatinine has dropped by >20% without dialysis in previously non-oliguric patients
    c. ATG completed and still oliguric
    d. One week passed
4. Solumedrol/Prednisone according to usual protocol

Use Tacrolimus/MMF in higher immunological risk as above.

5. Living Related Kidney Transplants

Start immunosuppression at least 24-48 hours before surgery if possible. Patients receiving HLA identical kidneys can have CNIs minimized after six months.

6. Tacrolimus

Tacrolimus is also a calcineurin inhibitor (CNI) in the same class as CyA. It is considered to be a more potent immunosuppressive and has a slightly different side-effect profile. In particular, it is associated with more new onset post transplant diabetes mellitus and has more GI side-effects, but less cosmetic side-effects, hypertension and gout. It is used in the following situations:

1) High Immunological Risk Patients
2) Steroid-Resistant and Vascular Rejection
3) Where intolerable CyA side effects are present. These are either neurological or severe gum hyperplasia or hirsutes.
4) When rejection occurs in the face of adequate blood concentrations of CyA.

Tacrolimus is started in a dose of 0.15 mg/kg/day administered in divided doses twice daily. Initial post transplant trough levels are 8-12ng/mL in the first 3 months, tapering to 4-10ng/mL thereafter.
A once daily formulation (*Advagraf*) is also available – daily starting dose is the same.
As a cost saving option, patients may be switched to a Ketoconazole combination with Tacrolimus once Isoniazid prophylaxis has been stopped, usually at 1 year post transplantation. The usual Tacrolimus dose reduction is about 50% when used with Ketoconazole 100mg bd. This strategy needs good patient understanding and close follow-up, otherwise risk of rejection/toxicity is not insignificant.

7. mTOR inhibitors

Sirolimus and Everolimus are mTOR inhibitors which are used predominantly in patients who have calcineurin inhibitor (CNI) side effects – predominantly CNI-induced nephrotoxicity - or the less common haemolytic uraemic syndrome (HUS) that may complicate calcineurin inhibitor use. Post-transplant Lymphoproliferative Disorder (PTLD) and Kaposi's Sarcoma are other uncommon indications for Sirolimus[5], though consideration should be given to switching to this class of immunosuppressives for any malignancy.
The starting dose of Sirolimus is 6mg daily, then 3 to 4mg daily (depending on patient size) with doses adjusted to trough levels (5-10ng/ml) – check after a *minimum* of 1 week (ideally 10-14 days) because of its long half-life (about 62 hours).
mTOR inhibitors should be used with caution in patients with proteinuria of >500mg/day, as this may be exacerbated, and not attempted if proteinuria equivalent to>1g/day. For those with proteinuria 500mg-1g/day, start on an ACE-I/ARB first - ARBs (eg. Losartan 50 to 100mg daily) are preferred because of a reported higher than normal rate of angio-oedema in patients on mTOR inhibitors in combination with an ACE-I[9]. Persistent proteinuria of >1-2g/day should prompt consideration for stopping the mTOR inhibitor.
Everolimus is an alternative drug in the same class with a shorter half-life. The starting dose is usually 0.75mg bd. Aim for trough levels between 3-8ng/mL.
Although not our routine practice currently, if/when mTOR inhibitors are used in combination with a CNI (more safety data with Everolimus), CNI trough levels should be low to avoid nephrotoxicity ie. cyclosporine trough levels of 50-100ng/mL or tacrolimus 3-5ng/mL. This combination can be used from the time of graft implantation and would mean that an anti-metabolite should not be used.

8. Mycophenolates

Mycophenolate, as a Mofetil (MMF) eg.*Cellcept/Mycocept* or an enteric coated sodium salt (MPS) *Myfortic*, is used as an anti-metabolite alternative to AZA. It is considered to be more potent than AZA and is non-nephrotoxic. It causes less bone marrow suppression than AZA, but has more GI side-effects (gastritis/diarrhoea). Indications are:

1) AZA is contra-indicated:
    a. gout requiring treatment with Allopurinol (interacts with AZA)
2) hepatotoxicity (usually cholestatic picture ie. raised Alk Phos/GGT) – exclude other causes firstHigh risk of rejection (see above)
3) Rejection on adequate doses of AZA and CNI
4) Steroid resistant rejection (see below)
5) Immunological cause for renal disease eg. SLE, rapidly progressive GN

The starting dose of MMF is 1g bd and MPS 720mg bd (MMF 500mg = MPS 360mg). Patients using Tacrolimus may be able to be weaned to lower doses later. For those experiencing GI side-effects or other MPA side-effects, consider switching to an alternative preparation of Mycophenolate (MMF to *Myfortic*), splitting the dose to 3 or

even 4x daily, reducing the dose (increased risk of rejection), or switching to AZA if not contraindicated.
The optimal dose of MMF with CyA is 1.5g bd (MMF entero-hepatic circulation which occurs in combination with Tacro is inhibited by CyA), but cost and side-effect profile will usually limit this to 1g bd.
Therapeutic Drug Monitoring (TDM) may also be used for MMF monitoring when over- or under-immunosuppression is clinically suspected, in which case aim for:

- Trough levels of:
  - 1.0-3.5mg/l for patients on CyA
  - 1.9-4.0mg/l for patients on Tacrolimus
- AUC levels of 30 to 60mg.h/L

If *Myfortic* is used, TDM is not recommended as it is a slow-release preparation.
NB. Mycophenolates are teratogenic, so women of child-bearing age should use AZA, or use adequate contraception if a Mycophenolate is strongly indicated.

9. Rituximab

Rituximab is a chimeric anti-CD20 monoclonal antibody that leads to B cell depletion. It is not licensed for use in renal transplantation but is in widespread use in ABO blood group incompatible transplantation. It is an effective treatment for post-transplant lymphoproliferative disorder, and is also used in both HLA antibody incompatible renal transplantation and the treatment of acute rejection. Recent evidence suggests rituximab may prevent the development of chronic antibody mediated rejection. The mechanisms underlying its effects are likely to relate both to long-term effects on plasma cell development and to the impact on B cell modulation of T cell responses.

Rituximab as a single dose (375mg/m^2) should be used in the following circumstances:

1. When we are planning to desensitize a patient for a living donor transplant (they usually have a flow or rarely CDC positive crossmatch, or are ABO incompatible). It should be given at least 2 to 4 weeks before transplantation, or
2. Immediately after a course of treatment of ABMR with PLEX/IVIG

10. Renal Biopsy

The role of renal biopsy in the management of immunosuppression is very important. If renal function is declining, a renal biopsy should performed to confirm the reason for this to enable a rational therapeutic decision to be made. The threshold for doing a biopsy will depend on how old the graft is and what has happened previously, but should not be delayed beyond a 50% rise from the baseline/best sCreatinine, and may be done well before this eg. 25% elevation above best, if thought necessary.
Even in patients with a well functioning graft, we strongly advise a "protocol" renal biopsy at 6 months post transplantation, but within 3 months of transplantation in patients who needed desensitization or have a DSA. This is to exclude sub-clinical rejection and to look for other complications eg. CNI nephrotoxicity.
An ultrasound of the graft kidney should first be done to exclude obstruction. Also examination of the urine should be done to exclude rejection/infection. Occasionally lymphocytes are identified on microscopy (with methylene blue staining), indicating probable rejection.
If the decline in graft kidney function is due to CNI toxicity, then the therapy of choice is an mTOR inhibitor, unless the patient has significant proteinuria on an ACE-I/ARB(>1g/day), in which case consider minimizing/stopping CNI and treating the patient

with a Mycophenolate and Prednisone only. This strategy does though carry increased risk of rejection.
In the event that rejection is the underlying problem then pulsed steroids and conversion to tacrolimus (if on CyA) and/or changing from AZA to MMF would be indicated.
Thus many patients, except those with the diagnosis of Chronic Allograft Nephropathy(CAN) [also called Interstitial Fibrosis Tubular Atrophy (IFTA-NOS)], or those with CNI induced HUS, will have histological indications for the use of either tacrolimus or an mTOR inhibitor, though even in these cases a change of immunosuppression may be beneficial.
The histological diagnosis of rejection and CNI toxicity is well described and defined: obliterative arteriolopathy with eccentric hyalinosis, ischemic collapse or scarring of the glomeruli, vacuolization of the tubules, and focal areas of tubular atrophy and interstitial fibrosis, producing a picture of "striped" fibrosis. These changes have though been described in patients who have never been on a CNI.
Other possible diagnoses on biopsy include rejection (Cellular, Humoral or both - see latest Banff Classification), recurrent or de novo disease esp. GNs, BK virus nephropathy, diabetic nephropathy, transplant glomerulopathy (usually a marker of antibody-mediated rejection) and many others.
Note: Renal biopsy may be impossible because of clinical contra-indications and treatment decisions may occasionally be taken on other criteria.

11. Treatment of acute rejection

11.1 Acute cellular rejection may be diagnosed clinically or histologically ie. with a renal biopsy - this is the method of choice. It is treated in the first instance with methylprednisolone (*Solumedrol*) in bolus doses. The standard protocol is 500 mg IVI daily for 3 days followed by a "skip" day followed by a 4th dose of Solumedrol. The doses may occasionally be adjusted downward for clinical indications, and outpatient therapy invariably omits the 4th dose. Baseline immunosuppression should be increased - ie. increase dose/levels of CyA if relatively low before, or change to from CyA to Tacrolimus, or AZA to Mycophenolate (for borderline rejection), or both if rejection severe/vascular.

11.2 Steroid Resistant and Vascular Rejection

In the event that the rejection fails to respond to pulsed steroids or vascular rejection is diagnosed on biopsy, ATG should be administered. The ATG regimen is as outlined for "high risk" patients, but at a higher cumulative dose ie. Fresenius-ATG 3-4mg/kg/day - usually 200mg daily - *or* Thymoglubuline 1.5mg/kg/day - rounded off to nearest 25mg - for 7-10 days. In addition, these patients should be changed to Tacrolimus if on CyA, and to MMF if on AZA. The anti-metabolite may be stopped until the white cell count recovers to >$4x10^9$/L, but this is at the discretion of the treating doctor. If the patient has not been pulsed with Solumedrol, this should be done as per usual. Hydrocortisone 100mg IV and Phenergan 25mg IV should be co-administered with ATG which is given via a central line. Remember to start CMV prophylaxis for at least 8 weeks after ATG.

11.3 Acute Antibody Mediated Rejection (ABMR)

A similar but more intensive strategy (daily if possible) of plasma exchange and IVIG as is used for desensitization (PLEX+IVIG - see 3.2 above) is used to treat acute antibody mediated rejection (ABMR) - but extended to at least 5 sessions in total. In addition, the patient should receive pulsed Solumedrol if not administered within the last few days already and a single dose of Rituximab 375mg/m^2 after the 5th session of PLEX+IVIG.

12. Infectious Disease Prophylaxis/Treatment

Tuberculosis Prophylaxis - Isoniazid 300mg daily (plus pyridoxine 25mg daily) for 1 year in all patients, except those with abnormal LFTs. Patients with suspected active TB should obviously not be transplanted at all.

Pneumocystis Pneumonia prophylaxis - Cotrimoxazole 80/400mg (1 tablet) daily for 6 months in all patients on Tacrolimus/Mycophenolate combination and post ATG or Desensitization or treatment for ABMR.

CMV prophylaxis must be given for at least 3 months in all patients who are CMV IgG positive, and ideally for 6 months in:

1) patients who are CMV IgG negative receiving a transplant from a CMV positive donor.
2) patients who have received ATG induction or treatment

Ideally this should be in the form of oral valganciclovir 900mg daily[6] (dose adjusted for abnormal GFR, thus 450mg daily is usually sufficient), but IV ganciclovir 5mg/kg daily (dose adjusted for abnormal GFR) can be used.

Patients with CMV syndrome/disease should treated with IV Ganciclovir if moderate to severe disease, or oral Valganciclovir if mild disease, till CMV PCR negative[7]. The dose used should in general be double the prophylaxis dose (usually the same dose but given *twice* daily, dose adjusted for renal failure). Treatment continues until CMV negative on weekly laboratory testing, followed by secondary prophylaxis for 6 weeks thereafter. Immunosuppression should be decreased to prevent a recurrence.

Low levels of *asymptomatic* CMV viraemia, typically CMV PCR Viral Load<log3 copies/mL, can be 'treated' by lowering the dose of immunosuppression, in particular the anti-metabolite, and repeat testing/close follow up. Viral Load>log3 copies/mL should be treated even if asymptomatic. Routine testing should be unnecessary for patients on Valgancyclovir prophylaxis and thus only needs to be done whenever CMV syndrome/disease is suspected off treatment or resistance is suspected (uncommon if dosed correctly).

Valganciclovir prophylaxis can be omitted in patients who are CMV IgG negative receiving a transplant from a CMV negative donor – this is a rare occurrence at GSH.

Influenza/Pneumonia Prophylaxis - Influenza A vaccination must be given annually and *Pneumovac* every 5 years.

Urinary Tract Infections are common and result in significant morbidity as well as increased risk of rejection. All symptomatic upper or lower urinary tract infections need to be treated for at least 10 days, as should asymptomatic urinary tract infections in the first 3 months post-transplant.

Antibiotics choice should be based on current or previous cultures, as available, and be guided by current hospital antibiotic protocols. Recurrent UTIs (≥3 in the last 12 months) need to be worked up for structural abnormalities with initially an ultrasound of the graft kidneys and native kidneys, ureters and bladder, and going on to do MCUG and urodynamic studies as needed, and in consultation with the urologists.

Routine Bloods on New Kidney Transplant Recipients (while still in-patients):
MONDAYS
CEU, FBC, PTT, INR
Trough CYCLOSPORINE / TACROLIMUS LEVELS
Ca, Mg, Phosphate
LFTs (Bili, ALT, Alk Phos, Alb)
MSU / MCS
Urine Protein:Creatinine Ratio
TUESDAYS
CEU
WEDNESDAYS
CEU, FBC
THURSDAYS
CEU
Trough CYCLOSPORINE / TACROLIMUS & MPA LEVELS
FRIDAYS
CEU, FBC
SATURDAYS
CEU
SUNDAYS
CEU

ALL RECIPIENTS SHOULD HAVE A RENOGRAM ON DAY 1 POST-TRANSPLANT, UNLESS TOO ILL/NEEDING URGENT PROCEDURE

NO BLOODS ON DONORS POST-OPERATIVELY UNLESS INDICATED.

A separate schedule of routine investigations to be done post discharge in the clinic is available.

13. Monitoring Patients in Transplant Clinic

BP checked at every visit – aim for <140/90 and ideally <130/80mmHg (no evidence). All antihypertensives can be used, though ACE-I +/- ARBs should be used early if proteinuria is present. A dramatic fall in GFR with ACE-I/ARBs should prompt investigation into Graft Renal Artery Stenosis (Doppler US +/- CT Angiography), as should declining GFR in a patient with difficult to control BP with a bruit. This complication is more common in grafts from living donors. An asymptomatic bruit can be watched, as this is a not uncommon finding.
Common problems in the transplant clinic include anaemia, post-transplant erythrocytosis, malignancies, osteoporosis, obesity, gout, new-onset diabetes after transplantation (NODAT) and many others. Treatment guidelines for all of these are beyond the scope of this protocol.

14. Drug interactions

Since the CNIs and the mTOR inhibitors are metabolized by hepatic cytochrome P450 3A enzyme system, a variety of important drug interactions with drugs that either affect or are metabolized by these enzymes can occur. Some are listed below, with common one's highlighted, but this list is by no means complete, so always check when prescribing a new drug. A useful website is http://reference.medscape.com/drug-interactionchecker?cid=med

Increase CNI/mTOR blood concentrations	Decrease CNI/mTOR blood concentrations
Diltiazem	Nafcillin
Nicardipine	Rifabutin
Verapamil	**Rifampin**
**Fluconazole**	Carbamazepine
Itraconazole	Phenobarbital
**Ketoconazole**	**Phenytoin**
**Clarithromycin**	Octreotide
**Erythromycin**	Ticlopidine
Lansoprazole	Orlistat
Cimetidine	**St. John's Wort**
Methylprednisolone	
Allopurinol	
Bromocriptine	
Metoclopramide	
Colchicine	
Amiodarone	
Danazole	
**Grapefruit juice**	

When starting patients on Rifampicin, the dose of the CNI/mTORi often needs to be increased 3 to 5 times - remember to check levels regularly in the following weeks and adjust accordingly. Also remember to double the dose of prednisone.
Remember the important AZA/Allopurinol interaction - without reducing the AZA dose by 66 to 75%, severe bone-marrow suppression can occur, so use MMF rather than AZA with Allopurinol (unless MMF is contra-indicated).

15. Cost of a lost renal allograft

A renal allograft enables a patient to be rehabilitated and to lead a normal life. Graft failure results in either the death or in the return to dialysis of that patient. Return to dialysis is by no means guaranteed, and depends on the patient's current medical, social and psychological status, as well as current availability.

Dr Zunaid Barday
Renal Unit & Transplant Unit, Groote Schuur Hospital
October 2017

References:

1. Tacrolimus and cyclosporine efficacy in high-risk kidney transplantation on behalf of the European Multicentre Tacrolimus (FK506) Renal Study Group. I.A.Hauser; H.-N.Neumayer. Transpl Int (1998) 11 [Suppl 1]: S73±S77.

2. Tacrolimus versus ciclosporin as primary immunosuppression for kidney transplant recipients: meta-analysis and meta-regression of randomised trial data. Angela C Webster, Rebecca C Woodroffe, Rod S Taylor, Jeremy R Chapman, Jonathan C Craig. BMJ, doi:10.1136/bmj.38569.471007.AE (published 12 September 2005).

3. Efficacy of Mycophenolate Mofetil Versus Azathioprine After Renal Transplantation: A Systematic Review. K. Wang, H. Zhang, Y. Li, Q. Wei, H. Li, Y. Yang and Y. Lu. Transplantation Proceedings, 36, 2071-2072 (2004)

4. Desensitization in HLA-Incompatible Kidney Recipients and Survival. Robert A. Montgomery, Bonnie E. Lonze, Karen E. King, Edward S. Kraus, Lauren M. Kucirka, Jayme E. Locke, Daniel S. Warren, Christopher E. Simpkins, Nabil N. Dagher, Andrew L. Singer, Andrea A. Zachary, and Dorry L. Segev. N Engl J Med 2011;365:318-26.

5. Anticancer Effect of Sirolimus in Renal Allograft Recipients With De Novo Malignancies. Transplantation Proceedings , Volume 39 , Issue 9 , Pages 2736 - 2739 M . Boratyńska , E . Wątorek , D . Smolska , D . Patrzałek , M . Klinger

6. Paya C, Humar A, Dominguez E, Washburn K, Blumberg E, Alexander B, Freeman R, Heaton N, Pescovitz MD, Valganciclovir Solid Organ Transplant Study Group. Efficacy and safety of valganciclovir vs. oral ganciclovir for prevention of cytomegalovirus disease in solid organ transplant recipients. Am J Transplant 2004 Apr;4(4):611-20.

7. Andre C. Kalil, MD; Josh Levitsky, MD; Elizabeth Lyden, MS; Julie Stoner, PhD; and Alison G. Freifeld, MD. Meta-Analysis: The Efficacy of Strategies To Prevent Organ Disease by Cytomegalovirus in Solid Organ Transplant Recipients. *Ann Intern Med.* 2005; 143:870-880.

8. Kidney Disease Improving Global Outcomes (KDIGO) Clinical Practice Guidelines for the Care of Kidney Transplant Recipients. American Journal of Transplantation 2009: 9 (Suppl 3)

9. Increased Incidence of Angioedema with ACE Inhibitors in Combination with mTOR Inhibitors in Kidney Transplant Recipients. Michael Duerr, Petra Glander, Fritz Diekmann, Duska Dragun, Hans-H. Neumayer, and Klemens Budde. Clin J Am Soc Nephrol. 2010 April; 5(4): 703-708

GSH Renal and Transplant Units
Kidney Transplant Protocol Summary June 2017

Solumedrol 500mg day 1, 250mg day2, 125mg day3, plus Prednisone 20mg/day. Wean Prednisone by 5mg/day every 2 weeks

<u>1.Low Risk (HLA identical) Patients</u>

CyA 3mg/kg bd (max. initial dose 300mg bd), AZA 2mg/kg/day (max. dose 150mg daily), only reduce dose if side-effects eg, WCC<4.0x10^9/L

Target trough CYA levels -200-300ng/mL in first 3 months
-150-250ng/mL 3 months till 1 year
-75-200ng/mL thereafter (watch for big decreases in CYA levels)

<u>2.Intermediate Immunological Risk Patients (all patients not Low or High Risk)</u>

Basiliximab(*Simulect*) 20mg IV on day 0 and day 4
CyA and AZA as above.

<u>3.High Risk Patients</u> <u>(previous transplant rejected, high PRA(>30%), DSA)</u>

ATG (aim for total dose Fresenius ATG to be 9mg/kg - usually 200mg daily for 3-5 days) + Tacrolimus 0.15mg/kg/day administered in divided doses twice daily, MMF 1g bd
Target trough Tacro levels are 8-12ng/mL in the first 3 months, tapering to 4-10ng/mL thereafter

Dose adjustments are done with following formula: New Dose = Target Level/Current Level x Current Dose

Hydrocortisone 100mg IV and Phenergan 25mg IV should be co-administered with ATG which is given via a central line

<u>DCD Recipients</u> - ATG + CyA(once graft functioning), AZA, Pred (doses as above), Use Tacrolimus/MMF in higher immunological risk as above.

<u>Rejection</u>

Pulse Medrol 500mg daily for 3 days (plus a 4th dose on day 5 if new transplant) and increase immunosuppression (increase dose of CNI if low, switch CNI (CyA to Tacro) and/or switch anti-metabolite (AZA to MMF) – latter option cheaper and should be tried first if rejection borderline

Steroid resistant/vascular rejection – ATG 3-4mg/kg/day (usually 200mg daily for 5-7 days) + switch to Tacrolimus + MMF

Antibody-Mediated Rejection – Pulse Medrol, plus 5 sessions of ideally daily plasmapharesis (1.5x plasma volume – usually 3-4L, replacing the pharesed fluid with 5% Albumin in a Balsol/Saline solution), plus IVIG 100mg/kg should be given at end of each PLEX session. Rituximab 375mg/m^2 post 5th PLEX/IVIG

<u>CNI toxicity</u>

Change from CNI to mTOR (sirolimus) unless proteinuria>1g/day on an ACE-I, then minimizing/stopping CNI and switch AZA to MMF is the better alternative

<u>Infectious Disease Prophylaxis/Treatment</u>

<u>Tuberculosis Prophylaxis</u> - Isoniazid 300mg daily (plus pyridoxine 25mg daily) for 1 year in all patients, except those with abnormal LFTs

<u>Pneumocystis Pneumonia prophylaxis</u> – Cotrimoxazole 80/400mg daily for 6 months in all patients on Tacrolimus/Mycophenolate combination and post ATG

<u>CMV prophylaxis</u> must be given for at least 3 months in all patients who are CMV IgG positive, and ideally for 6 months in:

1) patients who are CMV IgG negative receiving a transplant from a CMV positive donor
2) patients who have received ATG induction or treatment

	eGFR ml/min	Prophylaxis	Treatment
Ganciclovir IV	≥70	5mg/kg/dose daily	5mg/kg/dose bd
	50-69	2.5mg/kg/dose daily	2.5mg/kg/dose bd
	25-49	1.25mg/kg/dose daily	2.5mg/kg/dose daily
	10-24	0.625mg/kg/dose daily	1.25mg/kg/dose daily
	<10	0.625mg/kg/dose 3x/wk (post HD)	1.25mg/kg/dose 3x/wk (post HD)
Valgancyclovir PO	≥60	900mg/day	900mg bd
	40-59	450mg/day	450mg bd
	25-39	450mg every 2 days (or 225mg/d)*	450mg daily
	10-24	450mg twice weekly (or 125mg/d)*	450mg every 2 days (or 225mg/d)*
	<10	100mg* 3x/wk after dialysis	200mg* 3x/wk after dialysis

*For doses of less than 450mg, an oral solution is available. Treatment continues until CMV negative on weekly laboratory testing, followed by secondary prophylaxis for 4-6 weeks thereafter.
